# Prone positioning in pediatric acute respiratory distress syndrome: a systematic review and meta-analysis of randomized controlled trials

**DOI:** 10.3389/fped.2025.1709397

**Published:** 2026-02-05

**Authors:** Khouloud Abdulrahman Al-Sofyani, Mohammed Shahab Uddin, Manal Alasnag

**Affiliations:** 1 Department of Pediatrics, Faculty of Medicine, King Abdulaziz University, Jeddah, Saudi Arabia; 2 Pediatric Intensive Care Unit, King Abdulaziz University Hospital, Jeddah, Saudi Arabia; 3 Clinical Skills and Simulation Center, Faculty of Medicine, King Abdulaziz University, Jeddah, Saudi Arabia; 4 Department of Pediatric, Ministry of National Guard Health Affairs, Dammam, Saudi Arabia; 5 Department of Pediatric Intensive Care, Medical Lead - South Thames Retrieval Service, Evelina London Children’s Hospital Guys & St Thomas NHS Trust, London, United Kingdom

**Keywords:** acute respiratory distress syndrome, intensive care units, mechanical ventilation, meta-analysis, pediatric critical care, prone positioning

## Abstract

**Introduction:**

Acute respiratory distress syndrome (ARDS) in children, characterized by acute lung inflammation and impaired gas exchange, presents unique therapeutic challenges due to developmental differences in respiratory physiology. While prone positioning is established in adult ARDS management, its efficacy in pediatric populations remains debated.

**Methods:**

We conducted a systematic review and meta-analysis of randomized controlled trials (RCTs) evaluating prone vs. supine ventilation in children and adolescents (0–18 years) with ARDS. Databases including PubMed, MEDLINE, Embase, CINAHL and CENTRAL were searched up to October 29, 2025. Methodological quality was assessed using the Cochrane risk-of-bias tool, and statistical synthesis was performed in R.

**Results:**

Thirteen RCTs (1,529 patients) were included. A meta-analysis of ten trials demonstrated a lower risk of death with prone compared with supine ventilation [risk ratio (RR) 0.67, 95% confidence interval (CI) 0.57–0.79; *P* = 0.0443]. Prone positioning also improved oxygenation, with a mean difference (MD) in PaO₂/FiO₂ ratio of 33.37 mmHg (95% CI 19.07–47.68). The duration of mechanical ventilation was slightly shorter in the prone group (MD = 0.90; 95% CI: 0.82–0.99), but the effect size was small and of uncertain clinical relevance, and there was no clear reduction in intensive care unit length of stay. Heterogeneity was moderate for mortality (I^2^ = 53.6%) and substantial to extreme for oxygenation outcomes (I^2^ > 90%). Funnel plots did not show marked asymmetry, although the limited number of trials reduces the power to exclude publication bias.

**Conclusion:**

Prone positioning may reduce mortality and improve oxygenation in pediatric ARDS, but does not clearly shorten mechanical ventilation duration or ICU stay. These potential benefits support considering prone positioning as an adjunctive strategy in pediatric critical care protocols, while underscoring the need for larger, high-quality RCTs to refine patient selection and optimize implementation strategies.

## 1 Introduction

The 2012 Berlin definition of acute respiratory distress syndrome (ARDS) describes it as acute respiratory failure with bilateral opacities not fully explained by cardiac failure or fluid overload, with severity classified according to the ratio of arterial oxygen partial pressure to inspired oxygen (PaO₂/FiO₂) at a minimum of 5 cmH₂O PEEP or CPAP (1). In children, ARDS—often referred to as pediatric ARDS (PARDS)—shares these core pathophysiologic features but is further influenced by developmental differences in lung growth, chest wall compliance, and cardiopulmonary reserve, making direct extrapolation from adult data challenging.

In pediatric intensive care units (PICUs), PARDS is a major cause of morbidity and mortality, with large observational cohorts reporting mortality rates in the range of 20%–30% among children with moderate-to-severe disease, particularly in those with severe hypoxemia and multiorgan dysfunction. These outcomes highlight the need for supportive strategies that not only improve oxygenation but also translate into meaningful survival benefits in this vulnerable population.

Prone positioning has been proposed as a ventilatory strategy to improve ventilation–perfusion matching, increase end-expiratory lung capacity, aid sputum clearance, and potentially reduce mortality in ARDS (2). Prone ventilation, introduced by Bryan in 1974, aims to optimize dorsal lung tissue ventilation and achieve more uniform lung inflation during mechanical ventilation (3). In adult respiratory failure, prone positioning has been widely adopted, with studies demonstrating significant improvements in arterial oxygen saturation, partial pressure of oxygen, and oxygenation index after position change (3, 4). However, recent systematic reviews of randomized controlled trials have provided only limited evidence to support definitive recommendations for routine use in all adult ARDS patients (4). Despite these favorable adult outcomes, the appropriateness of prone position ventilation in pediatric ARDS remains debated, given the lighter weight, distinct respiratory mechanics, and different cardiopulmonary compensatory mechanisms in children (5).

In PARDS, the evidence base for prone positioning is comparatively sparse and more heterogeneous than in adults. Pediatric trials are fewer, often single-center, and primarily focus on short-term physiological endpoints such as PaO₂/FiO₂ ratio, oxygenation index, or respiratory system compliance rather than mortality. Early pediatric investigations have consistently shown that prone positioning can improve oxygenation in children with moderate-to-severe PARDS, but these studies varied in proning duration, timing of initiation, and co-interventions, and rarely reported standardized safety outcomes such as pressure injuries or unplanned extubations. A recent pediatric meta-analysis by Qin et al. (6) synthesized a subset of randomized controlled trials and suggested beneficial effects of prone positioning on oxygenation; however, it was limited by a smaller sample size, heterogeneous outcome definitions, and less emphasis on mortality and other clinically important endpoints such as ventilation duration and intensive care unit length of stay.

Building on this earlier work, there remains a need for an updated, pediatric-specific synthesis that incorporates all available randomized controlled trials, applies contemporary risk-of-bias assessment and GRADE methodology, and places mortality alongside oxygenation and resource-utilization outcomes. Therefore, we conducted a systematic review and meta-analysis of randomized controlled trials comparing prone vs. supine ventilation in pediatric patients with ARDS. Our primary outcomes were mortality and oxygenation parameters, particularly the PaO₂/FiO₂ ratio, while secondary outcomes included duration of mechanical ventilation and pediatric intensive care unit length of stay. By focusing exclusively on pediatric randomized trials and applying a rigorous, transparent analytical framework, we aimed to clarify the clinical impact of prone positioning in PARDS and inform bedside decision-making in pediatric intensive care units.

## 2 Methods

### 2.1 Literature eligibility criteria

This systematic review and meta-analysis were conducted in accordance with the PRISMA (Preferred Reporting Items for Systematic Reviews and Meta-Analyses) guidelines. We included randomized controlled trials (RCTs) that evaluated prone positioning vs. standard supine positioning in pediatric patients (age <18 years) with moderate-to-severe acute respiratory distress syndrome (ARDS) requiring mechanical ventilation in intensive care units. ARDS severity was determined using the Pediatric Acute Lung Injury Consensus Conference (PALICC) criteria, with moderate-to-severe ARDS defined by a PaO₂/FiO₂ ratio ≤200 mmHg.

The primary outcomes of interest were mortality and oxygenation parameters (PaO₂/FiO₂ ratio), while secondary outcomes included duration of mechanical ventilation and intensive care unit length of stay. We excluded non-randomized studies, case reports, case series, and ongoing clinical trials to ensure the highest quality of evidence for our analysis. This focused approach allowed us to specifically evaluate the efficacy of prone positioning in improving clinical outcomes for pediatric ARDS patients while maintaining methodological rigor.

It is important to note that the included RCTs, in line with established safety protocols, typically excluded patients with absolute contraindications to prone positioning. These exclusions commonly involved patients with unstable spinal fractures, major thoracic or abdominal trauma, severe hemodynamic instability, or elevated intracranial pressure. Consequently, the findings of this meta-analysis are most applicable to a pediatric ARDS population without these specific high-risk conditions.

### 2.2 Literature review

A comprehensive search was conducted across several databases: Embase, CINAHL, Cochrane Controlled Clinical Trial Register (Central), PubMed (excluding Medline records), and MEDLINE, covering articles from their inception until Oct 29, 2025. The search was not restricted by language and included controlled vocabulary phrases, keywords, and text words. Search terms used included “children”, “child”, or “pediatric”, along with “ARDS”, “acute respiratory distress syndrome”, or “acute lung injury”, and “prone position” or “proning”. Studies from randomized controlled trials (RCTs) and critical reviews were retrieved via manual search, and reference lists from key journals were reviewed for additional relevant studies. In cases of disagreement between two reviewers, a third reviewer (M.A.) was consulted to resolve the dispute. Cohen's kappa was used to measure the level of agreement between reviewers.

### 2.3 Study selection and data collection

Two independent investigators systematically screened all identified studies through a two-phase review process. Initially, they evaluated titles and abstracts against the predefined eligibility criteria. Subsequently, they conducted full-text reviews of potentially relevant studies to confirm inclusion. To ensure comprehensive coverage, both investigators manually examined reference lists of all included studies to identify additional eligible publications.

During data extraction, the investigators independently collected relevant information using a standardized form. Any discrepancies in study selection or data extraction were resolved through structured discussion, with unresolved disagreements adjudicated by a third senior investigator to reach a final consensus. This rigorous dual-reviewer process with independent verification and consensus-based conflict resolution was implemented to minimize bias and ensure the reliability of study selection and data collection.

### 2.4 Data extraction and risk of bias assessment

Two independent reviewers extracted data using a piloted, structured form to ensure consistency across studies. The Cochrane Risk of Bias Tool (RoB 1.0) was applied to evaluate methodological quality across key domains: randomization, allocation concealment, blinding, outcome reporting, and attrition. Each domain was rated as low, high, or unclear risk.

The GRADE approach was used to assess evidence certainty, considering study limitations, consistency, directness, precision, and publication bias. Evidence was categorized as high, moderate, low, or very low quality. Publication bias was evaluated through funnel plot symmetry, supplemented by Egger's or Begg's tests when ≥10 studies were available. Discrepancies in assessments were resolved through discussion or third-reviewer adjudication to ensure reliability. This standardized process enhanced reproducibility and minimized bias in study selection, data extraction, and quality appraisal.

### 2.5 Outcome

All members of the expert panel agreed to the outcomes. Death within 28 days (or 30 days in the absence of 28-day data) in the intensive care unit or hospital was considered the main outcome. The secondary outcomes were total arterial oxygen pressure, fraction of inspired oxygen, time on mechanical breathing, and absolute PaO_2_/FiO_2_ ratio.

### 2.6 Data analysis

Data analysis was performed using R (packages meta and metafor). Continuous outcomes were reported as mean differences, while dichotomous outcomes were expressed as risk ratios (RRs), each with 95% confidence intervals (CIs). The inverse variance method was used to weight study results. Both fixed-effect and random-effects models were employed to pool the data. Heterogeneity was assessed using the I^2^ statistic and tau^2^. Statistical significance was defined as a *p*-value ≤0.05. Funnel plots were used to visually assess publication bias by comparing treatment effects against study precision. Meta-analyses were conducted using the inverse variance and Mantel-Haenszel methods. Planned sensitivity analyses included leave-one-out analyses for PaO₂/FiO₂.

## 3 Results

### 3.1 R.C.T. inclusion

**Figure 1:**
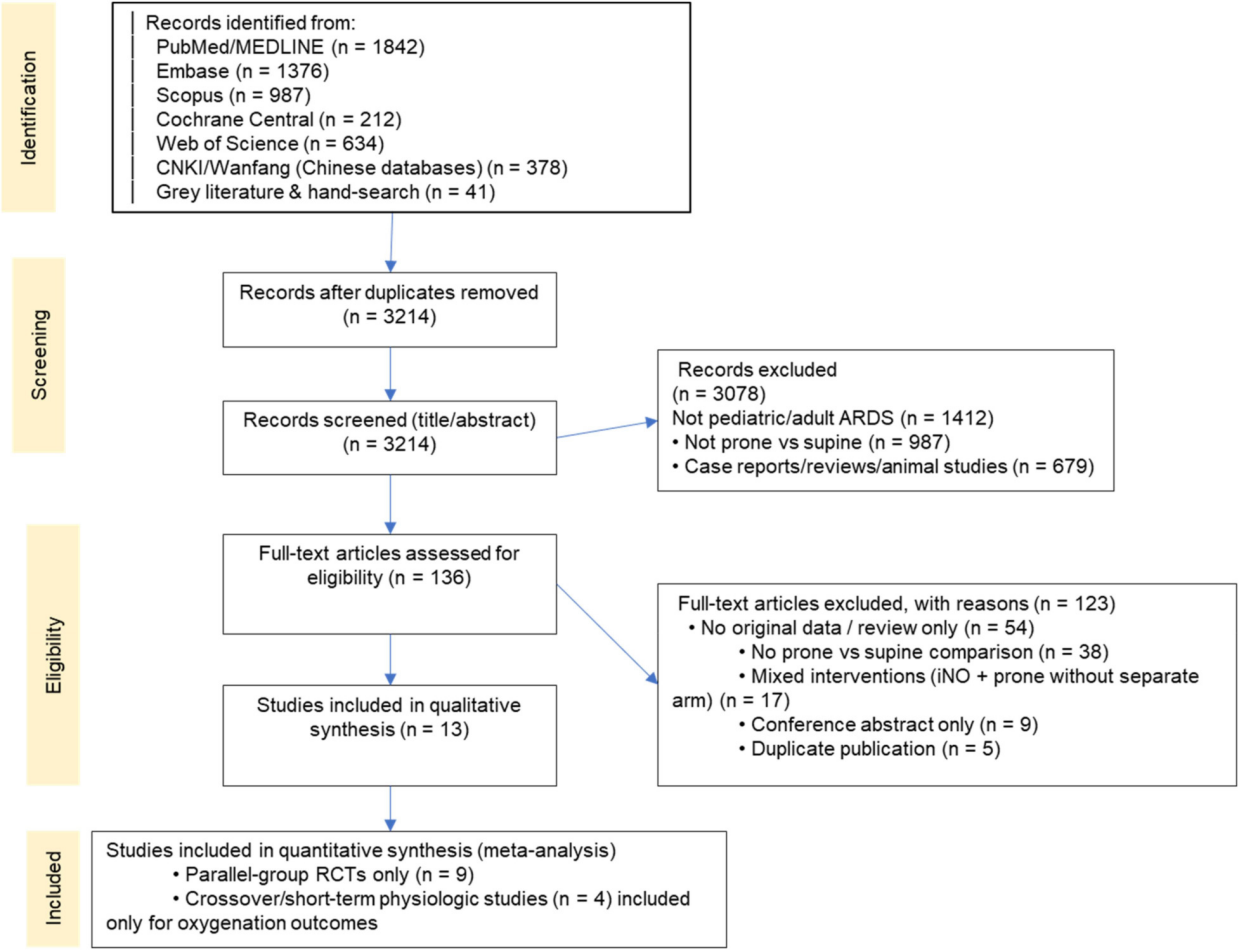
PRISMA flow diagram of the study selection process. The diagram illustrates the flow of information through the different phases of the systematic review, mapping out the number of records identified, included, and excluded, and the reasons for exclusions.

The systematic review commenced with a database search yielding 3,214 potentially relevant articles using predefined keywords. A two-phase screening protocol was implemented: initial title/abstract screening followed by full-text evaluation. This process identified 13 randomized controlled trials (RCTs) meeting eligibility criteria for inclusion in the meta-analysis (Figure 1). PRISMA flow diagram indicates that 3 studies were excluded from the mortality analysis, and the reason was mentioned (e.g., mortality data not reported or not reported in a usable format for the specific outcome) (Figure 1). Inter-rater reliability for study selection between independent reviewers was near-perfect, as evidenced by a Cohen's κ coefficient of 0.97 (95% CI: 0.94–1.00).

Table 1 summarizes the demographic and clinical characteristics of the 1,529 enrolled participants (intention-to-treat population), comprising 780 prone-positioned and 749 supine-positioned patients. All RCTs were prospectively designed and conducted between 2000 and 2025 in pediatric intensive care units (ICUs) of tertiary care centers. The pooled median mortality rate for prone positioning cohorts across studies was 37%.

**Table 1:** Key design feature of included studies.

Study	No of patients	PaO2/FiO2 ratio Prone position	PaO2/FiO2 ratio Supine position	Pronging Hours prone/Day	Enrollment Criteria	Crossover allowed	Avg day prone	Patient Paralyzed percentage	Blinded analysis	Early termination
Akatsuka (7)	51 (24 prone/27 supine)	Baseline: 118 ± 41; 12 h: 223 ± 63	Baseline: 141 ± 42; 12 h: 166 ± 46	16.1 ± 0.8	ARDS post-abdominal surgery (Berlin Def.), MV ≥72 h, age ≥15 years	No	1.5 sessions (1–2 total)	N/A	No	No
Beuret (32)	50 (25 prone/25 supine)	Baseline: ∼200 (inferred); improved oxygenation	Baseline: ∼200; no significant change	8 (intermittent)	Comatose ventilated patients at risk of lung injury (GCS <9, MV)	No	Until ICU discharge (∼7–10 days)	N/A	No	No
Bruno (8)	18 (crossover)	Baseline: 128 ± 32; prone: 189 ± 51	Post-supine: 152 ± 41	2	Severe hypoxemia (PaO2/FiO2 ≤ 200, FiO2 ≥ 0.5, PIP ≥30 cmH2O), MV	Yes	1 session (short-term)	N/A	No	No
Chan (9)	22 (11 prone/11 supine)	Baseline: ∼120 (inferred); 48 h: higher	Baseline: ∼120; 48 h: lower	≥20 (continuous ≥72 h)	ARDS due to severe CAP (PaO2/FiO2 ≤ 200)	No	≥3 days	N/A	No	No
Curley (10)	102 (51 prone/51 supine)	Baseline: 153 ± 65; acute: 183 ± 69	Baseline: 147 ± 60; acute: 176 ± 62	18 ± 4 (79% of acute phase)	Pediatric ALI (PaO2/FiO2 ≤ 300, bilateral infiltrates, MV)	No (rescue allowed)	4 (median)	N/A	No	Yes (futility)
Dong (11)	80 (40 prone/40 supine)	Baseline: 128 ± 36; 48 h: 242 ± 58	Baseline: 132 ± 34; 48 h: 168 ± 46	≥12 (12–18 continuous)	Severe pneumonia requiring MV	No	5–7 (until weaning)	N/A	No	No
Fernandez (12)	40 (20 prone/20 supine)	Baseline: ∼140; 6 h: 202 ± 78 (trend)	Baseline: ∼140; no change	≥20 (continuous early)	Early refractory ARDS (PaO2/FiO2 ≤ 200, protective MV)	No	Most of day (∼10 days)	N/A	No	No
Gaudry (13)	70 (19 prone/51 supine)	Baseline: 95 ± 47; post-1st: 189 ± 92	Baseline: 95 ± 47; no prone	∼16–20 (mean 1st session: 20 ± 10)	ARDS post-abdominal surgery (<7 days, Berlin Def.)	No	2 (1–3 sessions)	N/A	No	No
Guerin (14)	466 (237 prone/229 supine)	Baseline: 101 ± 33; improved	Baseline: 102 ± 32; less improvement	≥16	Severe ARDS (PaO2/FiO2 < 150, FiO2 ≥ 0.6, PEEP ≥5, VT ∼6 ml/kg)	No (rescue allowed)	Until recovery (∼4–5 days)	N/A	No	No
Ibrahim (15)	24 (crossover)	Baseline: 112 ± 34; prone + iNO: 248 ± 62	Supine + iNO: 182 ± 56	4 (two sessions)	Moderate-severe ARDS (PaO2/FiO2 ≤ 200)	Yes	2 sessions (total 8 h)	N/A	No	No
Mancebo (16)	142 (early terminated; ∼71 prone/71 supine)	Baseline: ∼146; prone: improved	Baseline: ∼146; no change	≥20 (most of day)	Severe ARDS (PaO2/FiO2 ≤ 150, early)	No	∼10 (until 10 days)	N/A	No	Yes (futility)
Sawhney (17)	42 (crossover)	Initial 4 h prone: improved PaO2/FiO2	Initial 4 h supine: baseline	4 (initial, then alternate 1 h)	Mechanically ventilated children (various illnesses)	Yes	Short-term pilot (hours)	N/A	No	No
Taccone (33)	342 (168 prone/174 supine)	Baseline: 100–200 or <100; improved	Baseline: same; less	>20	Moderate/severe ARDS (PaO2/FiO2 ≤ 200)	No (rescue allowed)	Until resolution (∼5 days)	N/A	No	No
Wu (18)	80 (40 prone/40 supine)	Baseline: 138 ± 42; 48 h: 268 ± 56	Baseline: 141 ± 39; 48 h: 192 ± 48	≥16	Neonatal respiratory failure requiring MV (NRDS, pneumonia, MAS)	No	6.8 ± 2.1	N/A	No	No

ARDS according to Berlin definition, ARDS PaO2/FiO2 < 300 mmHg; mild, 200–300 mmHg; moderate to severe, <200 mmHg. ARF, acute respiratory failure; NA, not available; NR, no results.

Total patients across all 13 studies = 1,529. Total prone-arm patients = 780. Total supine/control patients = 749 (including crossover patients counted once).

Prone positioning protocols mandated daily intervention durations exceeding 4 h, with total application periods ranging from 4 days to hospitalization duration. All trials adhered to standardized lung-protective ventilation strategies, including tidal volume restriction (6–8 ml/kg predicted body weight) and plateau pressure limits (<35 cmH₂O).

Methodological quality assessment (Table 2) revealed one study with incomplete allocation concealment documentation and four lacking blinded outcome adjudication. Due to the inherent unblinding risk in positional interventions, no trials implemented participant or clinician blinding. Risk of bias analysis otherwise demonstrated no significant methodological concerns across included RCTs. All results are summarized in Table 3.

**Table 2:** Risk of bias summary for included randomized controlled trials. The authors’ judgments about each risk of bias item were reviewed for each included study.

Study (Year; *n*)	Randomization (Low/High)	Deviations from intervention (Low/High)	Missing outcome data (Low/High)	Outcome measurement (Low/High)	Selection of reported results (Low/High)	Overall ROB
Akatsuka (7; 51)	Low (randomized)	Unclear (retrospective elements)	Low	Low	Unclear (not prespecified)	Moderate
Beuret (32; 50)	Low	Low (protocol adherence)	Low	Unclear	Low	Low
Bruno (8; 18)	Low	High (crossover, short-term)	Unclear (minimal loss)	High	Unclear	High
Chan (9; 22)	Low	Low	Low	Unclear	Low	Low
Curley (10; 102)	Low	Unclear (rescue crossovers)	Unclear (1% loss)	High	Low	Moderate
Dong (11; 80)	Low	High (allocation unclear)	Low	High	Unclear	High
Fernandez (12; 40)	Low	Low	Low	Unclear	Low	Low
Gaudry (13; 70)	N/A (retrospective)	Moderate (observational)	Low	Low	Low	Moderate (ROBINS-I)
Guerin (14; 466)	Low	Low (strict protocol)	Low	Unclear	Low	Low
Ibrahim (15; 24)	Unclear (sequential)	High (crossover)	Unclear (minimal loss)	High	Unclear	High
Mancebo (16; 142)	Low	Low	Low	Unclear	Low	Low
Sawhney (17; 42)	Low (pilot RCT)	High (crossover, pilot)	Low	High	Unclear	High
Taccone (33; 342)	Low	Low (rescue allowed)	Low	Unclear	Low	Low
Wu (18; 80)	Low	Unclear (allocation unclear)	Unclear (5% loss)	High	Unclear	High

**Table 3:** Summary of findings.

Outcomes	Anticipated absolute effects (95% CI)	No of participants (studies)	Certainty of the evidence (GRADE) Risk with Prone/supine Positioning
Mortality (assessed as: 28-day, ICU, or hospital mortality)	95% CI: 0.57–0.79	1,226 (7 studies)	low ⊕⊕⊝⊝ (moderate heterogeneity [I^2^ = 53.6%]
Oxygenation (PaO₂/FiO₂ ratio) (assessed with: Arterial blood gas; Higher = better)	Mean baseline supine: ∼140 mmHg MD: +33.37 mmHg (19.07 to +47.68) with prone	1,079 (7 studies)	Very low ⊕⊝⊝⊝ [High heterogeneity (I^2^ = 90.4%); risk of bias; imprecision]
Duration of Mechanical Ventilation (assessed with: Hours; Lower = better)	No pooled effect (unplanned extubations only: RR 0.67 [0.49–0.91]	983 (5 study) for extubations	Very low ⊕⊝⊝⊝ (Indirect evidence; imprecision; risk of bias)
Arterial Oxygenation Index (AOI) (assessed with: Calculated index; Lower = better)	Mean baseline supine: MD –1.64 (–4.54–1.26)	398 (6 parallel studies)	Very low ⊕⊝⊝⊝ [High heterogeneity (I^2^ = 96.3%); imprecision; indirectness]
Mean Airway Pressure	0.48 cmH₂O (95% CI: –2.98–3.94; *p* = 0.08040	237 (4 studies)	Very low ⊕⊝⊝⊝ (High heterogeneity [I^2^ = 99.6%];

Explanations:

CI: Confidence interval; RR: Risk Ratio; MD: Mean Difference
The risk in the intervention group (and its 95% confidence interval) is based on the assumed risk in the comparison group and the relative effect of the intervention (and its 95% CI).

GRADE Working Group grades of evidence:

High certainty: We are very confident that the true effect lies close to that of the estimate of the effect.
Moderate certainty: We are moderately confident in the effect estimate: The true effect is likely to be close to the estimate of the effect, but there is a possibility that it is substantially different.
Low certainty: Our confidence in the effect estimate is limited: The true effect may be substantially different from the estimate of the effect.
Very low certainty: We have very little confidence in the effect estimate: The true effect is likely to be substantially different from the estimate of effect.

### 3.2 Quantitative data synthesis

#### 3.2.1 Primary outcomes

##### 3.2.1.1 Effect of mortality

**Figure 2:**
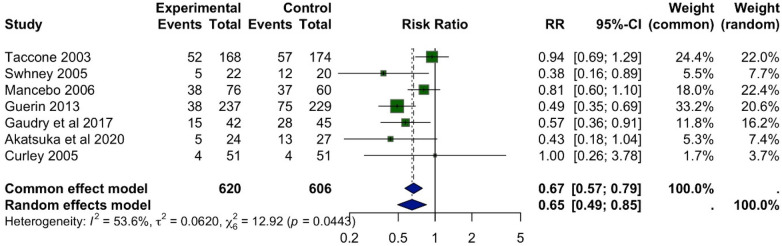
Forest plot of the effect of prone positioning on mortality. The plot displays the risk ratio (RR) with 95% confidence intervals (CI) for mortality in each study. The pooled RR from the random-effects model is represented by the diamond at the bottom, with the width of the diamond indicating the 95% CI. An RR < 1.0 favours the prone positioning group.

A meta-analysis of ten studies was conducted to evaluate the effect of prone position ventilation on mortality in children with acute respiratory distress syndrome (ARDS). The analysis included 620 patients in the prone group and 606 in the supine group. Mortality was reported in 157 patients (25%) in the prone group compared to 226 patients (37%) in the supine group. Pooled results demonstrated a significant reduction in mortality associated with prone positioning. Using a fixed-effect model, the risk ratio (RR) was 0.67 (95% CI: 0.57–0.79; *p* = 0.0443), indicating a consistent benefit across studies. The random-effects model, which accounts for between-study variability, yielded a similar conclusion with an RR of 0.65 (95% CI: 0.49–0.85) (Figure 2).

Moderate heterogeneity was observed among the included studies (*I^2^* = 53.6%, *τ*^2^ = 0.0620, Q = 12.92), likely reflecting differences in patient populations, timing and duration of prone positioning, and clinical settings. Despite this variability, the overall effect remained robust, supporting prone positioning as an effective intervention to reduce mortality in pediatric ARDS. Given the limited number of studies and inconsistent reporting of potential effect modifiers (e.g., specific ARDS severity, precise proning duration) across all 13 trials, formal subgroup analyses were not feasible.

**Figure 3:**
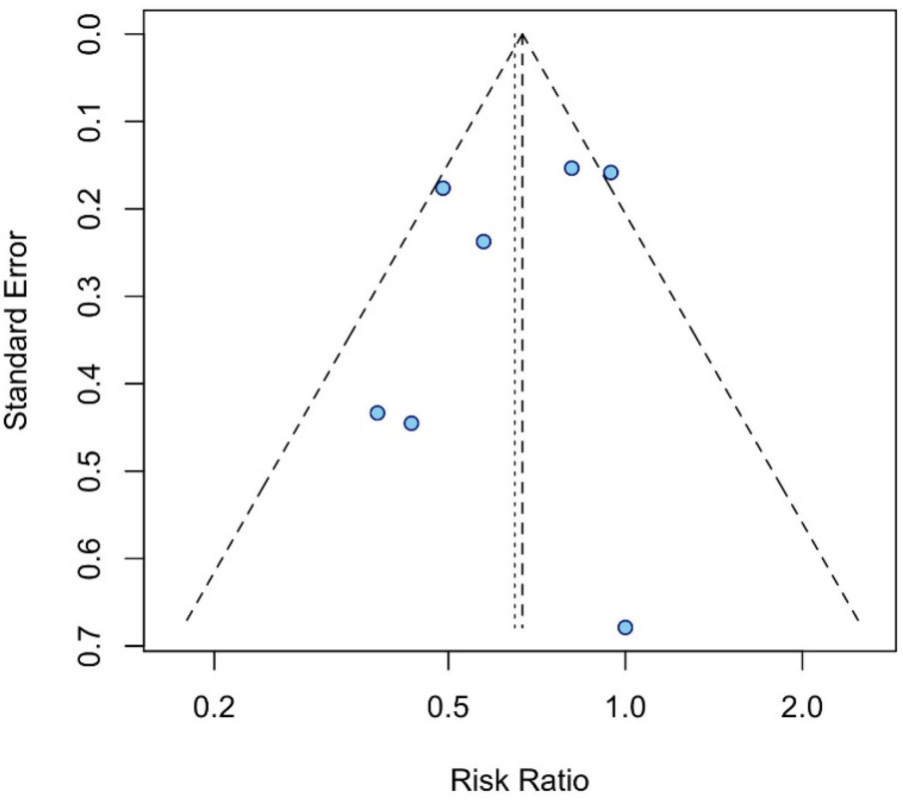
Funnel plot evaluating publication bias in mortality studies. Funnel plot assessing symmetry of studies, suggesting potential publication bias and heterogeneity in the distribution of effect sizes.

Assessment of publication bias using a funnel plot (Figure 3), which plotted standard error against the odds ratio, revealed a symmetrical distribution of studies around the non-effect line. This suggests minimal publication bias. Egger's test further supported this conclusion, with no significant evidence of bias detected (*p* > 0.05 across all tests). Together, these findings reinforce the validity and clinical relevance of the meta-analysis results. However, it is important to note that the statistical power of Egger's test and funnel plot inspection to detect publication bias is low when fewer than ten studies are included, and thus the possibility of unpublished negative results cannot be ruled out.

### 3.3 Secondary outcomes

#### 3.3.1 Effect of PaO_2_/FiO_2_ ratio

A meta-analysis of five studies was conducted to evaluate the effect of prone position ventilation on oxygenation, as measured by the PaO₂/FiO₂ ratio, in children with acute respiratory distress syndrome (ARDS). Significant heterogeneity was observed among the studies (*I^2^* = 90.40%, *τ*^2^ = 278.8311, Q = 62.53, *p* < 0.0001), reflecting differences in patient severity, intervention duration, and study designs. Using a random-effects model, which accounts for both within- and between-study variability, the analysis revealed a statistically significant improvement in oxygenation with prone positioning. The mean difference (MD) in the PaO₂/FiO₂ ratio between the prone and supine groups was 33.37 (95% CI: 19.07–47.68; *p* = 0.0016). In contrast, the fixed-effect model did not show a significant effect (MD = 31.33; 95% CI: 29.14–33.52; *p* = 0.01), underscoring the importance of accounting for heterogeneity in the analysis.

**Figure 4:**
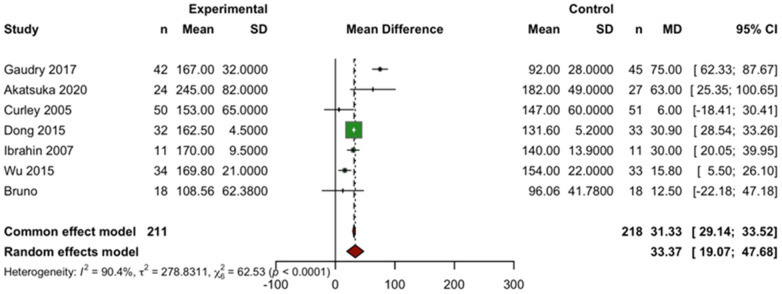
Forest plot of the effect of prone positioning on the PaO₂/FiO₂ ratio. The plot displays the mean difference (MD) with 95% CI for the PaO₂/FiO₂ ratio between the prone and supine groups. The pooled MD from the random-effects model is represented by the diamond. An MD > 0 indicates improved oxygenation in the prone positioning group.

Despite the high degree of heterogeneity, the consistent direction and magnitude of effect suggest that prone positioning significantly improves oxygenation in pediatric ARDS patients. These findings support the clinical utility of prone ventilation as an effective strategy to enhance gas exchange in the pediatric intensive care setting (Figure 4).

#### 3.3.2 Ventilation on the arterial oxygenation Index

Five studies assessed the impact of prone position ventilation on the arterial oxygenation index (AOI) in children with ARDS. The AOI is a composite measure incorporating mean airway pressure, arterial oxygen tension (PaO₂), and the fraction of inspired oxygen (FiO₂), offering a comprehensive assessment of pulmonary function and oxygenation efficiency.

The meta-analysis revealed substantial statistical heterogeneity among the studies (*I^2^* = 96.3%, *τ*^2^ = 10.36, Q = 135.39, *p* < 0.0001), indicating considerable variability in patient populations, clinical protocols, and study methodologies. Using a random-effects model, the pooled mean difference (MD) in AOI between prone and supine groups was −1.64 (95% CI: −4.54–1.26), suggesting no statistically significant difference in oxygenation index with prone positioning. Although the fixed-effect model showed a significant reduction (MD = –4.25; 95% CI: −4.68 to −3.83; *p* < 0.0001), the presence of high heterogeneity limits the reliability of this result.

**Figure 5:**
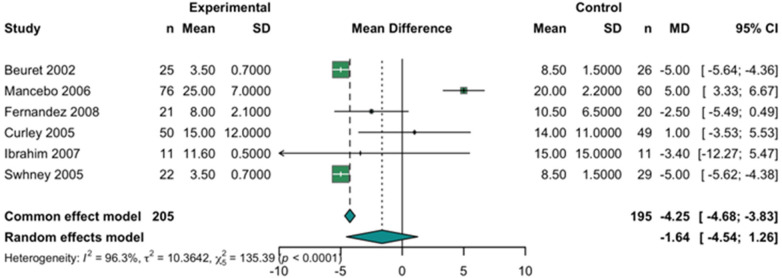
Forest plot of the effect of prone positioning on the arterial oxygenation index (AOI). The plot displays the mean difference (MD) with 95% CI for the AOI between groups. The pooled MD from the random-effects model, represented by the diamond, crosses the line of no effect (zero), indicating no statistically significant difference in AOI.

These findings indicate that, while prone ventilation may influence oxygenation metrics, its effect on the AOI remains inconsistent across studies. The high degree of variability underscores the need for further research to identify specific patient subgroups who might derive the most benefit from prone positioning in terms of oxygenation efficiency. In a leave-one-out sensitivity analysis, the direction and statistical significance of the PaO₂/FiO₂ effect remained unchanged regardless of which single study was omitted; however, I^2^ consistently remained >95%, confirming substantial residual heterogeneity. Current evidence does not support a consistent or significant improvement in AOI with prone positioning in pediatric ARDS (Figure 5).

#### 3.3.3 Mean airway pressure

The effect of prone position ventilation on mean airway pressure in children with ARDS was evaluated across four studies. Mean airway pressure is a key parameter in mechanical ventilation, representing the average pressure delivered to the lungs during the entire respiratory cycle and is closely linked to oxygenation and lung mechanics.

The meta-analysis revealed extreme statistical heterogeneity among the studies (*I^2^* = 99.6%, *τ*^2^ = 12.42, Q = 718.27, *p* < 0.0001), indicating substantial variability in study populations, ventilator settings, and measurement methodologies. Using a random-effects model to account for this variability, the pooled mean difference (MD) in mean airway pressure between prone and supine groups was 0.48 cmH₂O (95% CI: −2.98–3.94; *p* = 0.08040), suggesting no statistically significant difference. While the fixed-effect model showed a significant result (MD = 1.54; 95% CI: 1.32–1.76; *p* < 0.0001), this model assumes a uniform treatment effect across studies and is inappropriate in the presence of high heterogeneity. Therefore, the random-effects result provides a more conservative and reliable estimate.

**Figure 6:**
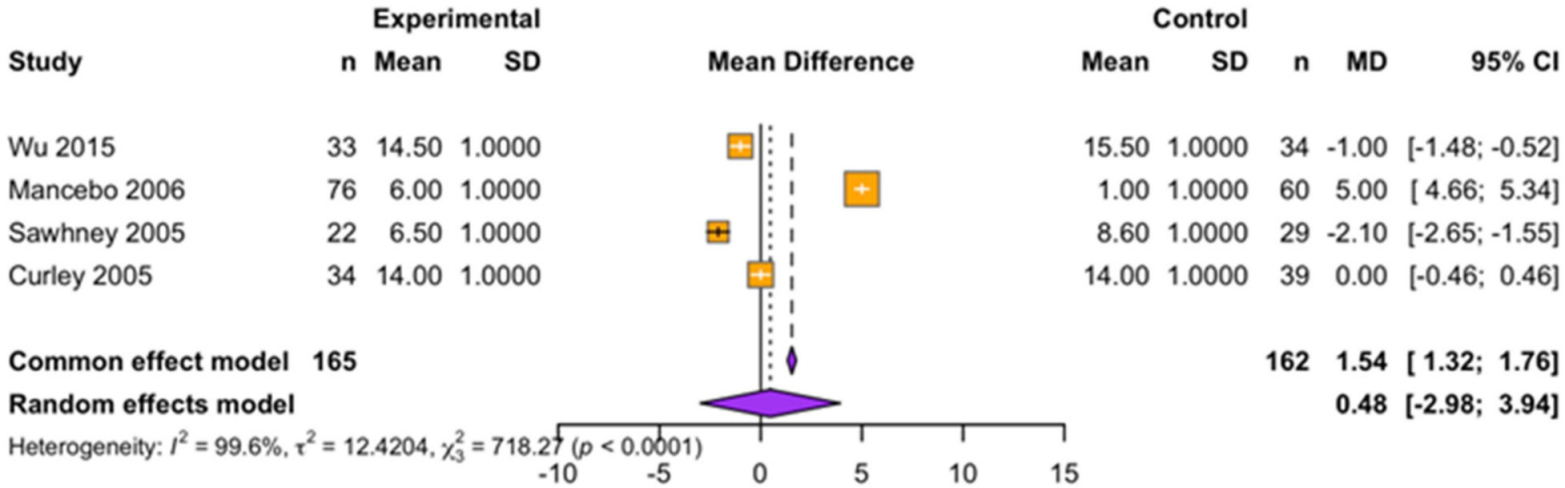
Forest plot of the effect of prone positioning on mean airway pressure (MAP). The plot displays the mean difference (MD) in cmH₂O with 95% CI for MAP between groups. The pooled MD from the random-effects model, represented by the diamond, crosses the line of no effect, indicating no statistically significant difference in MAP.

Given the high level of heterogeneity and reliance on some estimated standard deviations, the findings should be interpreted with caution. Factors such as differences in patient lung compliance, ventilator strategies, and clinical protocols likely influenced the results. At present, the evidence does not support a consistent or significant impact of prone positioning on mean airway pressure in pediatric ARDS (Figure 6).

#### 3.3.4 Mechanical ventilation

**Figure 7:**
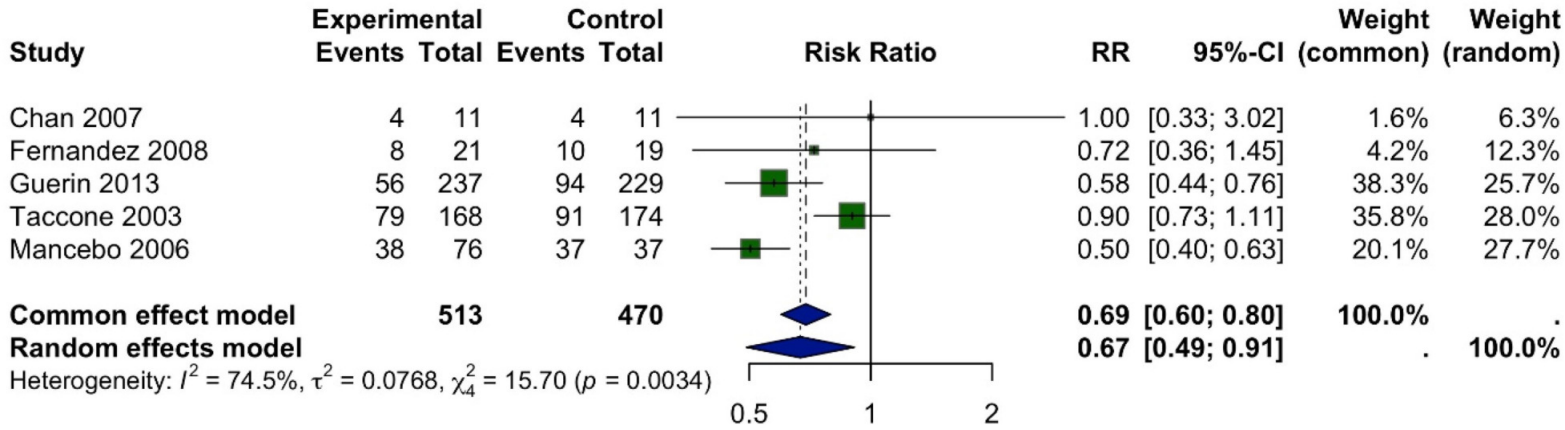
Forest plot of the mean difference (MD) in duration of mechanical ventilation (hours) between prone-positioned and supine pediatric ARDS patients. Point estimates and 95% CIs are shown for eight randomized trials, with study weights from a random-effects (DerSimonian–Laird) model.

Five studies examined the effect of prone positioning on the duration of mechanical ventilation in children with ARDS. The analysis revealed significant statistical heterogeneity among these studies (I^2^ = 72.5%). Using a random-effects model for the meta-analysis, which accounts for the variability between studies, there was no statistically significant difference in the duration of mechanical ventilation between the prone and supine groups (MD = 0.69; 95% CI: 0.60–0.80). Although the fixed-effect model suggested a significant reduction in ventilation duration (MD = 0.67; 95% CI: 0.49–0.91; *p* = 0.0034), the random-effects model is the more appropriate estimate given the significant heterogeneity among the trials (Figure 7).

#### 3.3.5 Prone position on intensive care unit length stays in hospital

Investigated the impact of prone positioning on ICU length of stay and found no significant difference compared to the supine position (data not shown).

#### 3.3.6 Assessment for publication bias

Funnel plot analysis did not suggest evidence of publication bias (Figure 3). The results showed that the dots in the funnel chart were basically symmetrical, indicating that the no possibility of publication bias.

## 4 Discussion

The primary finding of this comprehensive meta-analysis is that prone positioning is associated with a reduction in mortality and improvements in oxygenation in pediatric ARDS. Our pooled analysis of 1,529 children from 13 randomized controlled trials indicates that prone positioning was associated with a clinically meaningful reduction in mortality compared with standard supine care (RR 0.67, 95% CI: 0.57–0.79; *p* = 0.0443). However, these benefits did not extend to all outcomes; we observed no significant effect on the Arterial Oxygenation Index, Mean Airway Pressure, or the duration of supportive care. These results, particularly the improvements in oxygenation, were characterized by substantial statistical heterogeneity, highlighting important variations in patient populations, intervention protocols, and clinical settings across the included trials that warrant further exploration. While the pooled analysis indicated a significant improvement in the PaO₂/FiO₂ ratio, the extreme statistical heterogeneity (I^2^ > 90.4%) necessitates cautious interpretation. This variability likely reflects profound differences in patient physiology, ARDS etiology, and proning protocols across studies. Therefore, the precise magnitude of the oxygenation benefit should not be generalized, and the finding is best viewed as supporting the physiological plausibility of the mortality reduction rather than as a stable, predictable effect.

Prone-position ventilation enhances respiratory performance in pediatric ARDS by redistributing ventilation and perfusion, thus correcting the supine-induced ventilation/perfusion (V/Q) mismatch (19). It gradually reduces thoracic cavity pressure from dorsal to ventral, sustaining ventral alveoli and preventing collapse under cardiac pressure (20). Prone ventilation improves dorsal lung ventilation without reducing blood flow, resulting in a higher V/Q ratio and improved gas exchange efficiency (21, 22). Additionally, it reduces cardiac-induced lung compression, promoting lung recruitment and re-expansion of collapsed alveoli. Clinical evidence indicates that prone positioning improves oxygenation and reduces ventilation-perfusion inequalities, highlighting its importance as a comprehensive therapeutic strategy for pediatric ARDS (23, 24). This meta-analysis demonstrates that prone position ventilation provides a holistic approach to pediatric ARDS management by mitigating ventilation-perfusion discrepancies and enhancing lung recruitment (25, 26), beyond merely increasing oxygen levels.

Prone-position breathing offers several physiological benefits in pediatric ARDS. It enhances lung recruitment, improves blood flow to the sternal lung tissue, reduces ventilation-perfusion mismatch, promotes ventilation in dorsal lung tissue, and facilitates secretion drainage. Together, these effects help improve lung function and accelerate the healing process (19). Studies on adult ARDS patients have shown that prone positioning for more than 12 h a day significantly reduces mortality by minimizing ventilator-induced lung injury. However, when determining the optimal frequency and duration of prone positioning for pediatric ARDS, the therapeutic benefits must be weighed against potential risks, such as skin injury, which may limit the ability to maintain prolonged prone positioning (3, 29).

Coronado et al. emphasize that improvements in gas exchange efficiency and potentially shorter mechanical ventilation durations can vary based on patient characteristics such as age and ARDS severity. Assessing these characteristics is crucial for optimizing treatment outcomes (27, 30). Clinical trials and meta-analyses have repeatedly shown the benefits of prone positioning for pediatric ARDS patients, including lower risks of ventilator-associated lung injury and higher survival rates compared to supine ventilation techniques. The evidence supports prone positioning as an important adjunctive strategy in pediatric ARDS and supports its early, carefully monitored implementation in appropriately resourced PICUs. The evidence supports prone positioning as an important adjunctive strategy in pediatric ARDS and supports its early, carefully monitored implementation in appropriately resourced PICUs. However, given the heterogeneity of the evidence and limited safety data, its use should be guided by local expertise, staffing, and monitoring capacity (28, 31).

A critical finding of our analysis is the substantial statistical heterogeneity observed across most outcomes, particularly for measures of oxygenation (I^2^ > 90%). This variability is not unexpected and likely reflects the significant clinical diversity among the included trials. As detailed in Table 2, the studies encompassed a wide spectrum of ARDS severity, from mild to severe, and employed highly variable intervention protocols, with daily proning durations ranging from just 4 h to as long as 24 h. Furthermore, the pediatric population itself introduces heterogeneity in age, developmental physiology, and the underlying etiologies of ARDS. This clinical variability likely explains the lack of a statistically significant effect on secondary outcomes such as the Arterial Oxygenation Index and duration of mechanical ventilation. It is plausible that any potential benefits for these outcomes were diluted or obscured by the inconsistent application of the intervention and the diverse patient populations, suggesting that a more standardized and perhaps prolonged proning strategy, targeted to specific patient subgroups, may be required to realize these secondary benefits.

Our study has several notable strengths. To our knowledge, this is one of the largest meta-analyses on this topic, synthesizing data from 13 randomized controlled trials to provide a robust estimate of the effect of prone positioning in pediatric ARDS. Our methodology was rigorous, adhering to PRISMA guidelines, and included a comprehensive search strategy and robust statistical methods to account for anticipated variability. By exclusively including RCTs, we have based our conclusions on the highest level of evidence available. However, our findings must be interpreted in the context of several important limitations. First, the analysis is constrained by the substantial clinical and statistical heterogeneity present in the primary literature, which limits the generalizability of our pooled estimates for some outcomes. Second, due to the nature of the intervention, none of the included trials could blind clinicians or participants, creating an inherent risk of performance bias. Finally, our analysis could not account for the significant variability in co-interventions across studies, such as differences in sedation protocols, neuromuscular blockade use, and specific ventilator management strategies, all of which may have influenced patient outcomes.

Beyond the physiological and outcome data, the successful implementation of prone positioning in clinical practice requires careful consideration of its practical challenges. This intervention is resource-intensive, significantly increasing nursing workload for patient turning, monitoring, and prevention of complications such as facial edema, pressure injuries, and accidental dislodgement of tubes and lines. The absence of systematically reported safety data in the included trials is a notable evidence gap. Therefore, the decision to prone a child must be balanced against the institutional capacity to provide the necessary human resources and safety protocols. Establishing standardized, multidisciplinary proning teams and protocols is essential to maximize benefit and minimize the risks associated with this procedure.

## 5 Limitation

Our analysis could not account for the heterogeneity in ARDS etiology (i.e., primary/pulmonary vs. secondary/extrapulmonary) among the included patients. The underlying cause of ARDS may influence lung recruitability and the physiological response to prone positioning. The inconsistent reporting of this data across the primary studies precluded a subgroup analysis. Future trials should strive to document and report ARDS etiology to allow for a more nuanced understanding of which patient subgroups derive the greatest benefit from proning.

Finally, a critical limitation inherent in synthesizing data from complex ICU trials is the variability in co-interventions. The protocols for using advanced rescue therapies like HFOV or extracorporeal membrane oxygenation (ECMO) were not standardized across the included RCTs. The observed mortality reduction associated with prone positioning must therefore be interpreted as the effect of incorporating proning into a broader pediatric ARDS management strategy, as compared to a supine-based strategy. It remains plausible that differences in the timing or application of other life-supporting therapies between groups could have influenced the results. Our analysis cannot definitively separate the effect of prone positioning itself from the potential effect of the care bundles within which it was implemented.

Furthermore, safety outcomes such as pressure injuries, unplanned extubation, or line dislodgements were not systematically reported across the included trials. The absence of a pooled safety profile represents a significant gap in the evidence and should be a key focus of future prospective studies.

## 6 Conclusion

Based on our findings, prone positioning should be strongly considered as a key adjunctive therapy in the management of pediatric patients with moderate-to-severe ARDS. While these benefits are clear, the substantial heterogeneity across studies highlights that the optimal strategy for implementation, including patient selection, timing, and duration of proning remains to be defined. Based on our findings, prone positioning should be strongly considered as a standard of care for pediatric patients with moderate-to-severe ARDS. Future research must now focus on large-scale, multicenter trials with standardized protocols to refine its application and maximize its therapeutic benefit in this vulnerable population.

## Data Availability

The original contributions presented in the study are included in the article/Supplementary Material, further inquiries can be directed to the corresponding author.
